# Thermal transport and anharmonic phonons in strained monolayer hexagonal boron nitride

**DOI:** 10.1038/srep43956

**Published:** 2017-03-06

**Authors:** Shasha Li, Yue Chen

**Affiliations:** 1 Department of Mechanical Engineering, The University of Hong Kong, Pokfulam Road, Hong Kong SAR, China

## Abstract

Thermal transport and phonon-phonon coupling in monolayer hexagonal boron nitride (h-BN) under equibiaxial strains are investigated from first principles. Phonon spectra at elevated temperatures have been calculated from perturbation theory using the third-order anharmonic force constants. The stiffening of the out-of-plane transverse acoustic mode (ZA) near the Brillouin zone center and the increase of acoustic phonon lifetimes are found to contribute to the dramatic increase of thermal transport in strained h-BN. The transverse optical mode (TO) at the K point, which was predicted to lead to mechanical failure of h-BN, is found to shift to lower frequencies at elevated temperatures under equibiaxial strains. The longitudinal and transverse acoustic modes exhibit broad phonon spectra under large strains in sharp contrast to the ZA mode, indicating strong in-plane phonon-phonon coupling.

Two dimensional (2D) hexagonal boron nitride (h-BN), which has a direct band gap of 5–6 eV, has attracted great attentions in recent years as a promising material for electronics and optoelectronics^1^,^2^,^3^. Hexagonal boron nitride may also be used as a substrate material for high quality graphene electronics because of its identical crystal structure with graphene^4^,^5^. The thermal conductivity of few-layer h-BN at room temperature was found to be in the range from 227 to 280 W/mK^6^,^7^, which is much smaller than that of graphene. The relatively low thermal conductivity of h-BN is a disadvantage for its potential applications in electronics, optoelectronics and substrates^1^,^2^,^3^,^4^,^5^. For bulk materials, thermal conductivity usually decreases under tensile strains; e.g., based on lattice dynamics calculations and equilibrium molecular dynamics (MD) simulations, the thermal conductivities of bulk silicon and diamond were found to decrease under tensions due to the reductions in group velocities and heat capacities^8^,^9^. Using lattice dynamics calculations and Boltzmann transport equation, the thermal conductivity of bulk Lennard-Jones argon was also predicted to decrease under tensile strains because of the decrease of phonon lifetimes and group velocities^8^. However, in contrast to generally reduced thermal conductivities of bulk materials under tensile strains due to the decrease of group velocities, phonon lifetimes or heat capacities^8^,^9^,^10^, it was found that the thermal conductivities of some 2D materials can be enhanced via strains. For example, the thermal conductivity of graphene is enhanced under strains^11^,^12^. The thermal conductivities of silicene and silicene nanoribbons were found to increase greatly and then fluctuate at an elevated value under strains due to the stiffening of the out-of-plane transverse acoustic mode (ZA) mode^13^. The thermal conductivity of single-layer phosphorene was also reported to enhance under a zigzag-oriented strain^14^. The thermal conductivity of uniaxially strained monolayer h-BN was recently studied using the Tersoff potential and it was found that the thermal conductivity increased initially and then decreased at further strains. The phenomenon was attributed to a competition between in-plane softening and flexural stiffening of the phonons^15^,^16^. In the present study, the thermal conductivity of monolayer h-BN is found to enhance more rapidly under equibiaxial strains comparing to previously reported values under uniaxial strains^15^,^16^. Phonon-phonon coupling of monolayer h-BN are investigated from first principles density functional theory calculations, which provide deeper insight into the strain effects on thermal transport. In addition to the increased group velocity of the ZA mode^15^,^16^, it is further found that the increased lifetimes of the acoustic phonon modes also play an important role in the enhancement of the thermal conductivity.

Lattice thermal transport is dominated by phonon-phonon coupling, which is also important for understanding the lattice stability of h-BN under equibiaxial strains. Lattice instability resulted from the softening of the transverse optical mode (TO) phonon mode at the K point was reported to dominate the mechanical failure of h-BN under equibiaxial strains^17^. As previous studies only focused on the lattice dynamics at T = 0 K, it is important to further investigate the effects of phonon-phonon coupling on the lattice stability. It was shown that the optical phonon modes obtained from classical MD simulations based on the Tersoff potential^18^ significantly deviated from experimental results^19^, making the Tersoff potential unsuitable for studying the phonon-phonon coupling. In the current work, the phonon spectra at elevated temperatures are obtained by calculating the temperature-dependent phonon self-energy, Σ_*λ*_(*ω*) = Δ_*λ*_(*ω*) − *i*Γ_*λ*_(*ω*), which measures the anharmonic phonon-phonon coupling; the real part Δ_*λ*_(*ω*) describes the frequency shift, and the imaginary part Γ_*λ*_(*ω*) describes the decay of phonon, which is inversely proportional to the phonon lifetime. The TO mode at the K point is found to depend significantly on temperature due to strong phonon-phonon coupling under large equibiaxial strains.

## Computational details

The harmonic phonon dispersions at 0 K are calculated using the finite displacement approach with Phonopy^20^. The anharmonic effects at elevated temperatures are obtained by calculating the third-order force constants with Phonon3py^21^. At a finite temperature of T, the imaginary part of phonon self-energy Γ_*λ*_(*ω*) for a specific phonon mode *λ* can be evaluated to the lowest order in many body perturbation theory^21^,


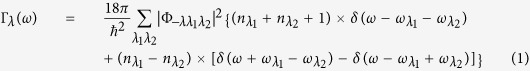


where *n*_*λ*_ is the Bose-Einstein occupation factor,


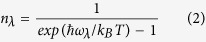


and the matrix element 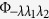 describes the interactions among three phonons *λ, λ*_1_ and *λ*_2_, which can be calculated from the third-order anharmonic force constants. The real part of self-energy, Δ_*λ*_(*ω*), can be calculated using the Kramers-Kronig relation.


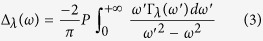


The power spectrum 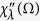 of a phonon mode *λ* can be calculated with^22^:


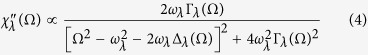


where *ω*_*λ*_ is the harmonic frequency of a specific phonon mode.

Lattice thermal conductivity is calculated by solving the linearized phonon Boltzmann equation (LBTE) using the single-mode relaxation-time (SMRT) method^21^,^23^:


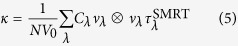


where *V*_0_ is the unit cell volume; the thickness of monolayer h-BN is taken as 0.333 nm, which is equal to the interplanar distance in bulk h-BN^24^. *ν*_*λ*_ and *C*_*λ*_ are the group velocity and heat capacity of a phonon mode *λ*, respectively. The single-mode relaxation-time, 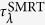, can be approximated by the phonon lifetime *τ*_*λ*_^21^, which is the reciprocal of the phonon linewidth 2Γ_*λ*_(*ω*_*λ*_).


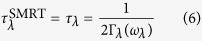


Supercells of 9 × 9 × 1 and 6 × 6 × 1 were used to obtain the second and third order force constants using the finite displacement approach^20^,^21^, respectively. Force constants were calculated based on density functional theory using Vienna Ab initio Simulation Package (VASP)^25^. The total energy tolerance was set to 10^−8^ eV. The projector augmented wave (PAW) method was applied and the exchange-correlation interactions were treated with the local density approximation (LDA) functional. The valence electronic states were expanded in plane wave basis sets with an energy cutoff of 520 eV. The Brillouin zone integrations were performed with 3 × 3 × 1 and 4 × 4 × 1 Monkhorst Pack grids^26^ for the 9 × 9 × 1 and 6 × 6 × 1 supercells, respectively. The equilibrium lattice parameter of monolayer h-BN obtained from our calculations is 2.488 Å, which is slightly smaller than the experimental value of 2.504 Å^27^. The underestimation of lattice constants is a well-known issue of LDA functional. A vacuum of 20 Å was applied to the h-BN supercells. The equibiaxial strain is defined as the nominal strain *ε*_*x*_ = *ε*_*y*_ = (*l*_*i*_ − *l*_0_)/*l*_0_, where *l*_*i*_ is the lattice constant under strains and *l*_0_ is the original lattice constant.

## Results and Discussions

**Figure 1:**
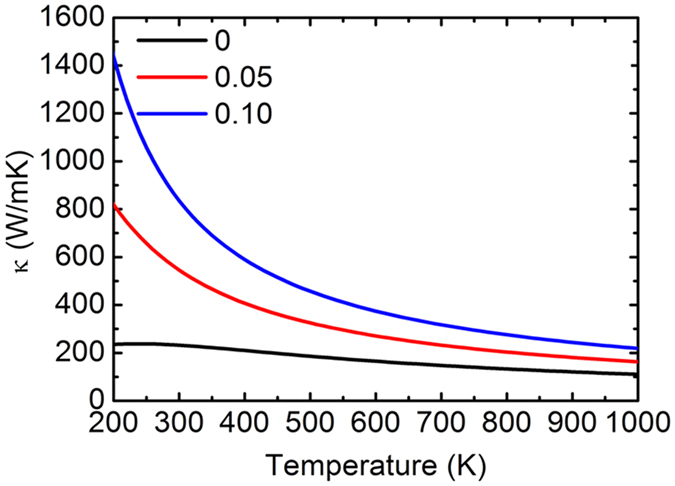
Thermal conductivity of monolayer h-BN as a function of temperature under strains of *ε*_*x*_ = *ε*_*y*_ = 0, 0.05 and 0.10.

Lattice thermal conductivity of monolayer h-BN with strains of *ε*_*x*_ = *ε*_*y*_ = 0, 0.05 and 0.10 are shown in Fig. 1. Our theoretical thermal conductivity of unstrained monolayer h-BN is 232 W/mK at 300 K, in good agreement with experimental value of about 250 W/mk for five-layer h-BN^6^. Contrary to the general reduction in thermal conductivities of bulk materials under tensile strains^8^,^9^,^10^, the thermal conductivity of monolayer h-BN increases significantly, with values of 546 and 835 W/mK at 300 K for *ε*_*x*_ = *ε*_*y*_ = 0.05 and 0.10, respectively. Strain-induced enhancements of thermal conductivities of 2D materials have also been reported previously; e.g. graphene^11^,^12^, silicene^13^, phosphorene^14^ and monolayer h-BN^15^,^16^. It is expected that the thermal conductivity of monolayer h-BN will start decreasing under very large strains due to the weakening of atomic interactions; however, very large strains are difficult to realize in practical applications. In line with previous studies using the Tersoff potential^15^,^16^, our results indicate that monolayer h-BN has a better heat dissipation performance under strains. Furthermore, equibiaxial strains are found to be more effective in increasing the thermal conductivity comparing to uniaxial strains^15^.

**Figure 2:**
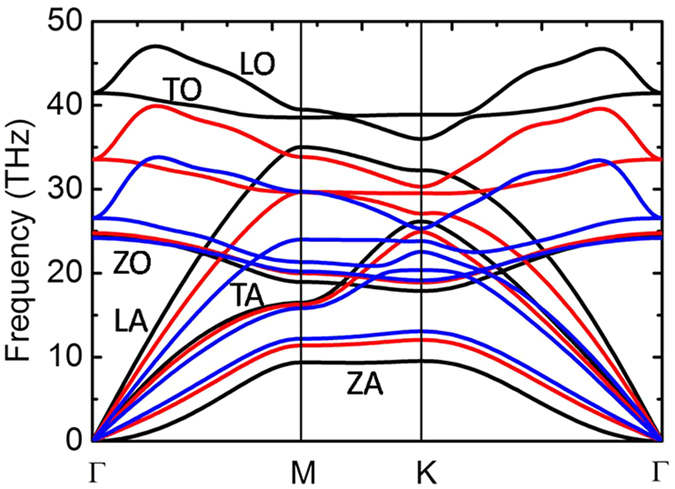
Phonon dispersions of monolayer h-BN with strains of *ε*_*x*_ = *ε*_y_ = 0 (black line), 0.05 (red line) and 0.10 (blue line) at T = 0 K.

In order to have deeper insight into the mechanisms that lead to an enhanced thermal conductivity, we have calculated the phonon dispersions at 0 K under different strains (see Fig. 2). The splitting between the longitudinal (LO) and transverse optical (TO) modes at the Γ point is not observed due to the 2D nature of monolayer h-BN^28^,^29^. In general, acoustic phonon modes contribute more to the lattice thermal conductivity because the group velocities of acoustic modes are larger than those of optical modes. It is seen from the phonon dispersions of h-BN (Fig. 2) that there are only minor changes in the group velocities of the optical modes under equibiaxial strains. On the other hand, softening of the transverse acoustic (TA) and the longitudinal acoustic (LA) modes is observed, indicating reduced group velocities. The ZA mode behaves greatly different from TA and LA modes, it exhibits a parabolic dispersion near the Γ point with low group velocity for unstrained monolayer h-BN, while under equibiaxial strains, the dispersion of the ZA mode is linearized and stiffened, resulting in a much higher group velocity that contributes to the enhancements of thermal conductivity. Although the above discussion is based on calculations at 0 K, similar behaviors of the different modes are also observed at elevated temperatures (see Supplementary Fig. S1). Our calculations suggest that, under equibiaxial strains, the stiffening of the ZA mode contributes to the enhanced thermal conductivity due to the enhanced group velocity.

**Figure 3:**
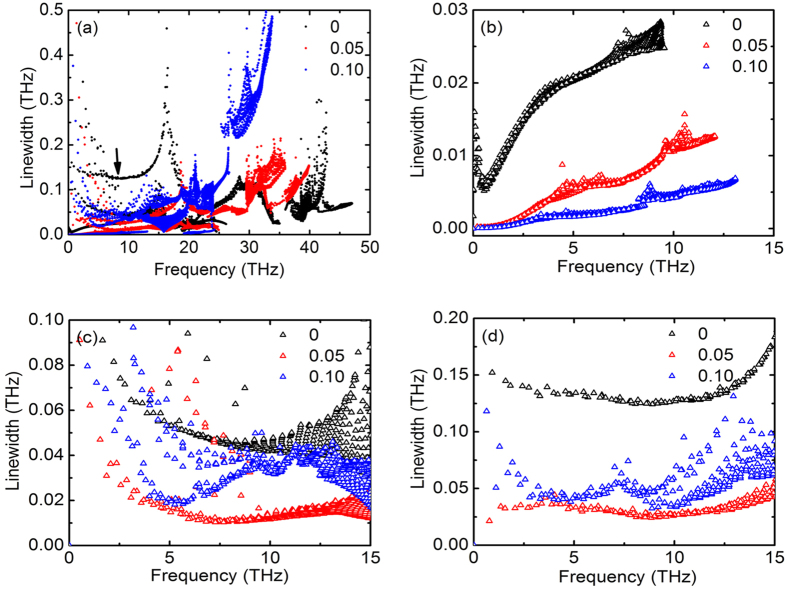
Phonon linewidth. (**a**) Phonon linewidths of all phonon modes in monolayer h-BN under strains of *ε*_*x*_ = *ε*_*y*_ = 0, 0.05 and 0.10 at 300 K. (**b**) Phonon linewidths of the ZA mode at the low frequency region. (**c**) Phonon linewidths of the TA mode at the low frequency region. (**d**) Phonon linewidths of the LA mode at the low frequency region.

In addition to the group velocities change caused by frequency shift under equibiaxial strains, we perform further analysis on the phonon lifetimes, the reciprocal of the phonon linewidths 2Γ_*λ*_(*ω*_*λ*_), at elevated temperature when phonon-phonon interactions become significant. The linewidths of different phonon modes at 300 K for strains of *ε*_*x*_ = *ε*_*y*_ = 0, 0.05 and 0.10 are given in Fig. 3a. It is seen that the linewidths of the optical modes in the high frequency region become larger under strains, indicating shorter phonon lifetimes originate from stronger phonon-phonon coupling. Considering the minor changes in the group velocities of the optical modes, the shorter lifetimes indicate a smaller contribution to the lattice thermal conductivity. Different from the optical modes, significantly reduced LA mode linewidths are observed under strains (see where the arrow points to), indicating longer LA phonon lifetimes. On the other hand, as we have discussed previously that the softening of the LA mode under strains leads to a reduced group velocity. Therefore, the overall contribution of the LA mode to the lattice thermal conductivity becomes non-obvious under strains. For detailed analysis, the phonon linewidths of ZA, TA, LA modes are given in Fig. 3b,c and d, respectively. It is found that, for unstrained monolayer h-BN, the linewidths of the ZA modes have the smallest values among the different acoustic phonons (i.e. the longest lifetimes), suggesting a large contribution from the ZA modes to the total thermal conductivity. The important role of the ZA mode in thermal transport has also been reported for other 2D materials; for example, Seol *et al*. attributed the lower thermal conductivity of supported graphene than that of suspended graphene to the strong interface-scattering of the ZA mode^30^, and they also demonstrated that the lattice thermal conductivity of graphene is dominated by the ZA mode through an exact numerical solution of the phonon Boltzmann equation^31^. As shown in Fig. 3b, the linewidths of the ZA modes decrease monotonously with increasing strains, contributing to the enhanced thermal conductivity when equibiaxial strain is below 0.10. The linewidths of the TA and LA modes change in more complicated manners; it is found that they firstly decrease and then increase under strains. The different strain-dependent behaviors of the acoustic phonon linewidths may be related to their different vibration directions; the TA and LA modes are in-plane vibrational modes, whereas the ZA mode is an out-of-plane vibrational mode. Considering the variations in phonon group velocities and lifetimes under equibiaxial strains, the contribution from the ZA mode to the lattice thermal conductivity becomes larger and the contributions from the optical modes become smaller as equibiaxial strain increases.

**Figure 4:**
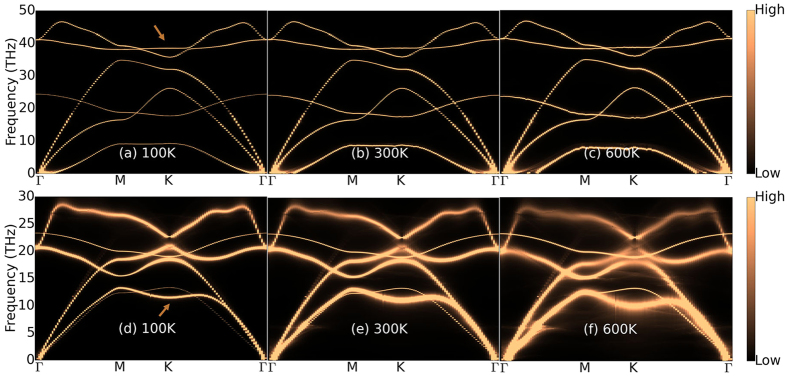
Phonon spectra of monolayer h-BN under equibiaxial strains of *ε*_*x*_ = *ε*_*y*_ = 0 (**a**,**b** and **c**) and 0.14 (**d**,**e** and **f**) at different temperatures. The arrows point to the TO mode at the K point.

As equibiaxial strain is very effective in increasing the thermal conductivity of monolayer h-BN, it is important that the system remains stable under large strains. The softening of the TO mode at the K point is believed to lead to the failure of h-BN before it reaches the limit of elastic stability^17^. The phonon dispersions of monolayer h-BN at 0 K have been calculated under different equibiaxial strains, and the softening of the TO mode at the K point is successfully reproduced. The equibiaxial strain at which the TO mode becomes unstable is *ε*_*x*_ = *ε*_*y*_ = 0.18 (see Supplementary Fig. S2), which is in agreement with previous report^17^. To better understand the phonon induced instability, we have further studied the phonon-phonon coupling effects at elevated temperatures. It is seen from the phonon spectra shown in Fig. 4 that, for unstrained h-BN, no obvious frequency shift of the TO mode is observed at elevated temperatures. On the other hand, the TO mode behaves differently when h-BN is under a strain of *ε*_*x*_ = *ε*_*y*_ = 0.14. Significant broadening and negative shift of the TO mode at the K point is observed as temperature increases, indicating strong phonon-phonon coupling in strained h-BN. The negative shift of the TO mode at elevated temperatures indicates a phonon induced lattice instability at a smaller strain comparing to the value previously predicted based on T = 0 K calculations^17^. Nonetheless, the equibiaxial strain for phonon induced instability is well above 0.10, which is the highest strain considered for thermal conductivity.

For unstrained monolayer h-BN, it is also found that the parabolic dispersion of the ZA mode near the Γ point becomes flatter as temperature increases, indicating a lower group velocity of the ZA mode near the zone center due to phonon coupling at elevated temperatures. For h-BN under a large equibiaxial strain, the LA and TA modes become very broad with rising temperature (see Fig. 4e and f), whereas the ZA mode remains very thin. In other words, in-plane phonon-phonon coupling is found to be much stronger than that of the out-of-plane phonons. Our calculations suggest that the ZA mode dominates the thermal transport in h-BN under large equibiaxial strains because of their much longer phonon lifetimes.

**Figure 5:**
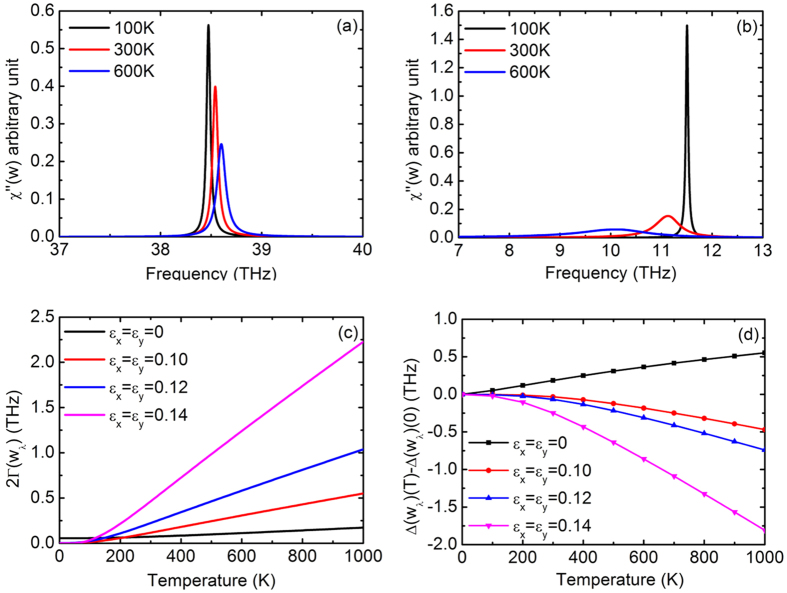
(**a**) Phonon spectra of the TO mode at the K point at different temperatures for unstrained monolayer h-BN. (**b**) Phonon spectra of the TO mode at the K point at different temperatures for *ε*_*x*_ = *ε*_*y*_ = 0.14. (**c**) Temperature-dependent linewidth of the TO mode at the K point for *ε*_*x*_ = *ε*_*y*_ = 0, 0.10, 0.12 and 0.14. (**d**) Temperature-dependent frequency shift of the TO mode at the K point for *ε*_*x*_ = *ε*_*y*_ = 0, 0.10, 0.12 and 0.14.

For detailed examination of the temperature effects on the TO mode at the K point, its phonon spectrum is given in Fig. 5a and b for strains of *ε*_*x*_ = *ε*_*y*_ = 0 and 0.14, respectively. It is seen that the TO mode phonon spectrum of unstrained h-BN shifts slightly to higher frequency as temperature increases. On the other hand, the phonon spectrum shifts to lower frequency at high temperatures when the system is under a strain of *ε*_*x*_ = *ε*_*y*_ = 0.14. Broadening of the spectrum is observed at high temperatures, and the effect of broadening becomes more significant in strained h-BN. The phonon linewidth 2Γ(*ω*_*λ*_) and the frequency shift of the TO mode under strains of *ε*_*x*_ = *ε*_*y*_ = 0, 0.10, 0.12 and 0.14 are shown as functions of temperature in Fig. 5c and d. For unstrained h-BN, the linewidth and the frequency shift are relatively small. The temperature effects become more significant with increasing equibiaxial strains, suggesting stronger phonon-phonon coupling.

## Conclusion

The thermal conductivity of monolayer h-BN under equibiaxial strains is investigated by solving the LBTE within the SMRT method. It is found that the thermal conductivity can be effectively enhanced under equibiaxial strains. The stiffening of the ZA phonon mode near the Brillouin zone center and their increased lifetimes are found to contribute to the enhanced lattice thermal conductivity. Phonon spectra at elevated temperatures have been calculated for detailed investigations of the strain effects on phonon-phonon coupling. For unstrained h-BN, the frequency of the TO mode at the K point is found to shift to higher frequency as temperature increases, while for the system under equibiaxial strains, the TO mode shifts to lower frequency, indicating that the phonon induced lattice instability in h-BN is accelerated at high temperatures. Strong in-plane phonon-phonon coupling is observed in heavily strained h-BN, where the phonon spectra of the LA and TA modes are largely broadened at elevated temperatures while that of the ZA mode remains very thin. The phonon-phonon coupling insight obtained in this work may provide a guideline for the strain engineering of the thermal transport in h-BN.

## Additional Information

**How to cite this article**: Li, S. and Chen, Y. Thermal transport and anharmonic phonons in strained monolayer hexagonal boron nitride. *Sci. Rep.* **7**, 43956; doi: 10.1038/srep43956 (2017).

**Publisher's note:** Springer Nature remains neutral with regard to jurisdictional claims in published maps and institutional affiliations.

## Supplementary Material

Supplementary Material
