# Supplementary material for: Thermal transport and anharmonic phonons in strained monolayer hexagonal boron nitride
Source: Sci Rep. 2017 Mar 6;7:43956. doi: 10.1038/srep43956 (PMC5338266; doi:10.1038/srep43956)
Supplement: Supplementary Material [file srep43956-s1.pdf]

Thermal transport and anharmonic phonons in strained monolayer hexagonal boron nitride

Shasha Li, Yue Chen [1†]

Department of Mechanical Engineering, The University of Hong Kong,

Pokfulam Road, Hong Kong SAR, China

Phonon spectra of monolayer h-BN at 300 K under equibiaxial strains of $\varepsilon_x = \varepsilon_y$ =0, 0.05 and 0.10 are given in Supplementary Figure S1. Similar to phonon dispersions at 0 K, the linearizaion and stiffening of ZA mode, and the softening of TA and LA modes under equibiaxial strains are also found at 300 K. In addition to the frequency shift, the change of phonon linewidths under different equibiaxial strains can also be observed. The linewidths of in-plane optical modes increase with increasing strain, indicating shorter phonon lifetimes due to stronger phonon coupling. The linewidths of the ZA modes decrease under strains, contributing to the enhanced thermal conductivity.

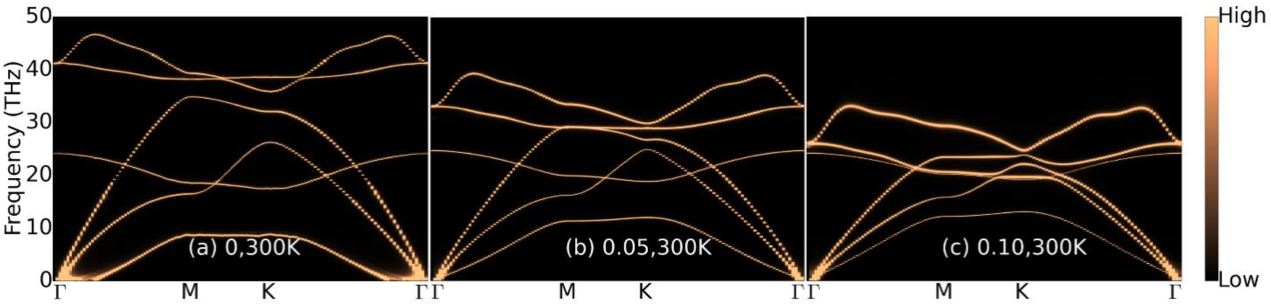

Supplementary Figure. S1: Phonon spectra at 300 K of monolayer h-BN under equibiaxial strains of (a) $\varepsilon_x = \varepsilon_y$ =0, (b) $\varepsilon_x = \varepsilon_y$ =0.05 and (c) $\varepsilon_x = \varepsilon_y$ =0.10.

Phonon dispersions of monolayer h-BN at 0 K under different equibiaxial strains were calculated, and the softening of the TO mode at the K point is found (see Supplementary Figure S2). It is seen that the TO mode becomes imaginary when $\varepsilon_x = \varepsilon_y$ =0.18.

1 * Corresponding author. E_mail: yuechen@hku.hk

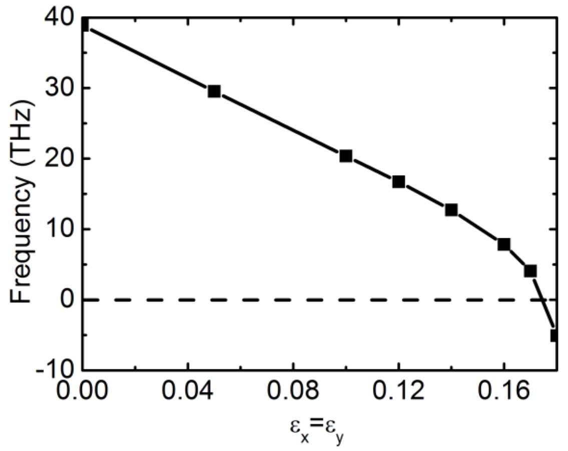

Supplementary Figure. S2: Frequency of the TO mode at the K point at 0 K as a function of equibiaxial strains. Negative values indicate imaginary frequencies.
